# Mucinous carcinoma in a male breast with skin ulcer: a case report

**DOI:** 10.3389/fonc.2025.1521704

**Published:** 2025-02-06

**Authors:** Xiaohui Lin, Tingting Liao, Yuting Yang, Jingzhi Zhou, Jie Ma

**Affiliations:** Department of Radiology, Shenzhen People’s Hospital, The Second Clinical Medical College of Jinan University, Shenzhen, China

**Keywords:** male breast tumor, magnetic resonance imaging, cutaneous mucinous carcinoma, breast mucinous carcinoma, skin ulcer

## Abstract

A case of pure mucinous carcinoma of the male breast presenting with skin ulceration was reported. The patient, a 67-year-old male, inadvertently discovered a subcutaneous mass with the size of a soybean near the areola on the right side of his chest. Pathological analysis identified the mass as mucinous adenocarcinoma. Differentiating between primary mucinous carcinoma of the skin and mucinous carcinoma of the breast was challenging due to their overlapping histological and immunohistochemical features. Ultimately, the tumor was diagnosed as pure mucinous carcinoma of the male breast based on the primary site and clinical history.

## Introduction

Male breast cancer is a rare disease, accounting for less than 1% of all breast cancer cases worldwide (1, 2). Mucinous carcinoma, also known as colloid or gelatinous carcinoma, is extremely rare in males. Differentiating between breast mucinous carcinoma and cutaneous mucinous carcinoma (e.g., primary mucinous degeneration of the skin or mucinous skin cancer) in males through radiological and histopathological examinations can be challenging, particularly when the skin is ulcerated. This study aimed to report a case of pure mucinous adenocarcinoma of the male breast with skin ulceration and review the clinical and imaging features of this exceptionally rare tumor, providing valuable information for the differential diagnosis of cutaneous mucinous carcinoma.

## Case presentation

### Patient’s information

The patient is a 67-year-old male who inadvertently discovered a subcutaneous nodule, about the size of a soybean, near the areola on the right side of his chest in 2020. The patient reported undergoing ultrasound and CT scans at a local hospital, although the specific details were unclear. The nodule was initially suspected to be a dermatofibroma and was left untreated. Recently, the patient noticed rapid growth of the nodule into a mass accompanied by skin erythema and ulceration. Seeking further treatment, he was admitted to the breast surgery department of our hospital on July 28, 2022. He had no family history of breast cancer or other malignancies.

### Clinical finding

**Figure 1:**
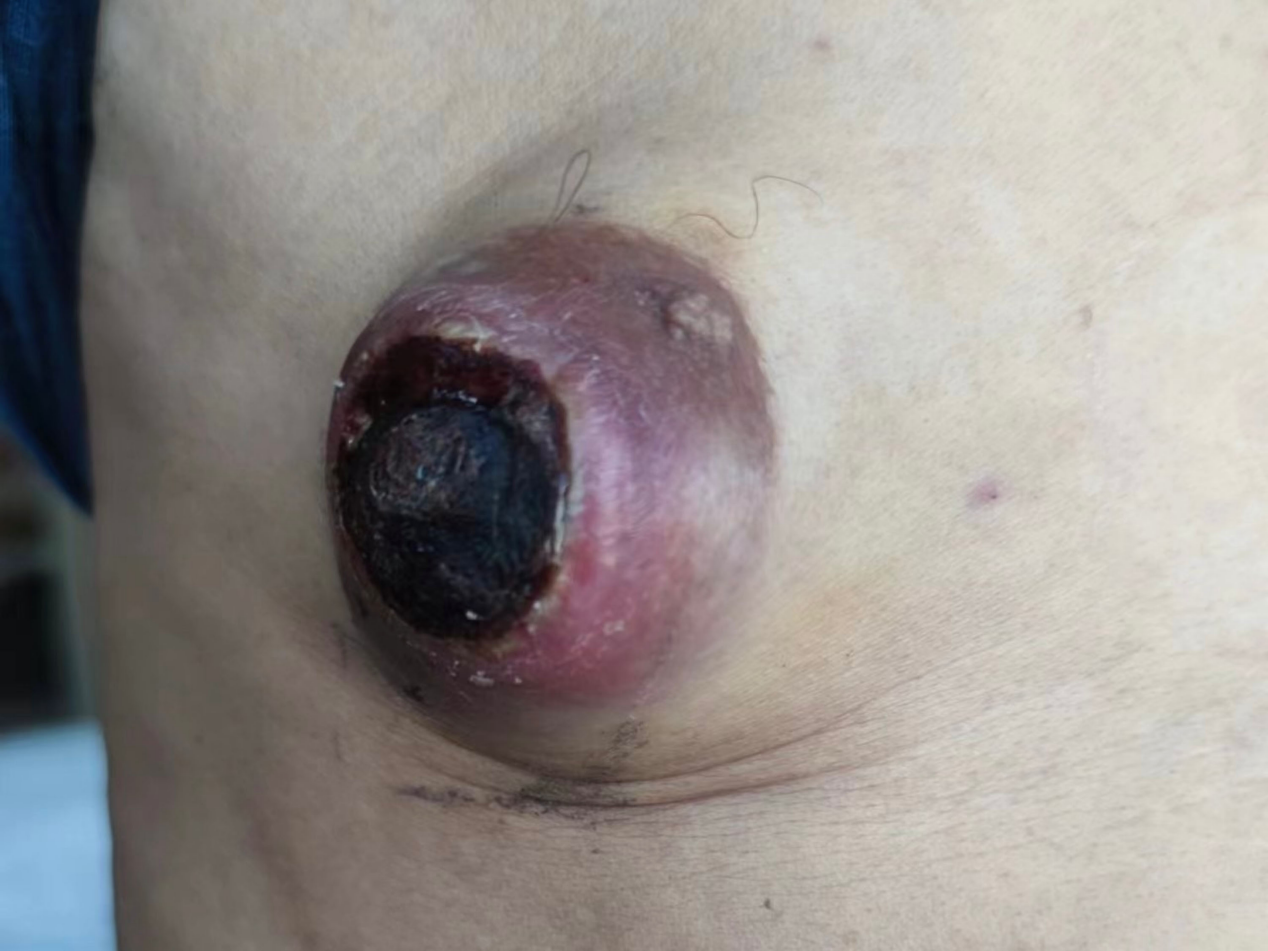
A mass on the right side of the male patient’s chest.

Physical examination revealed a large mass behind the nipple on the right side of his chest, about 5.0 cm × 4.0 cm in size, with an uneven surface, soft texture, unclear boundaries, poor mobility, and no adhesion to the chest wall. The surface skin was dark red and ulcerated (**Figure 1**). Enlarged lymph nodes, about 1.5 cm in size, were palpable in the right axilla.

### Imaging examinations

**Figure 2:**
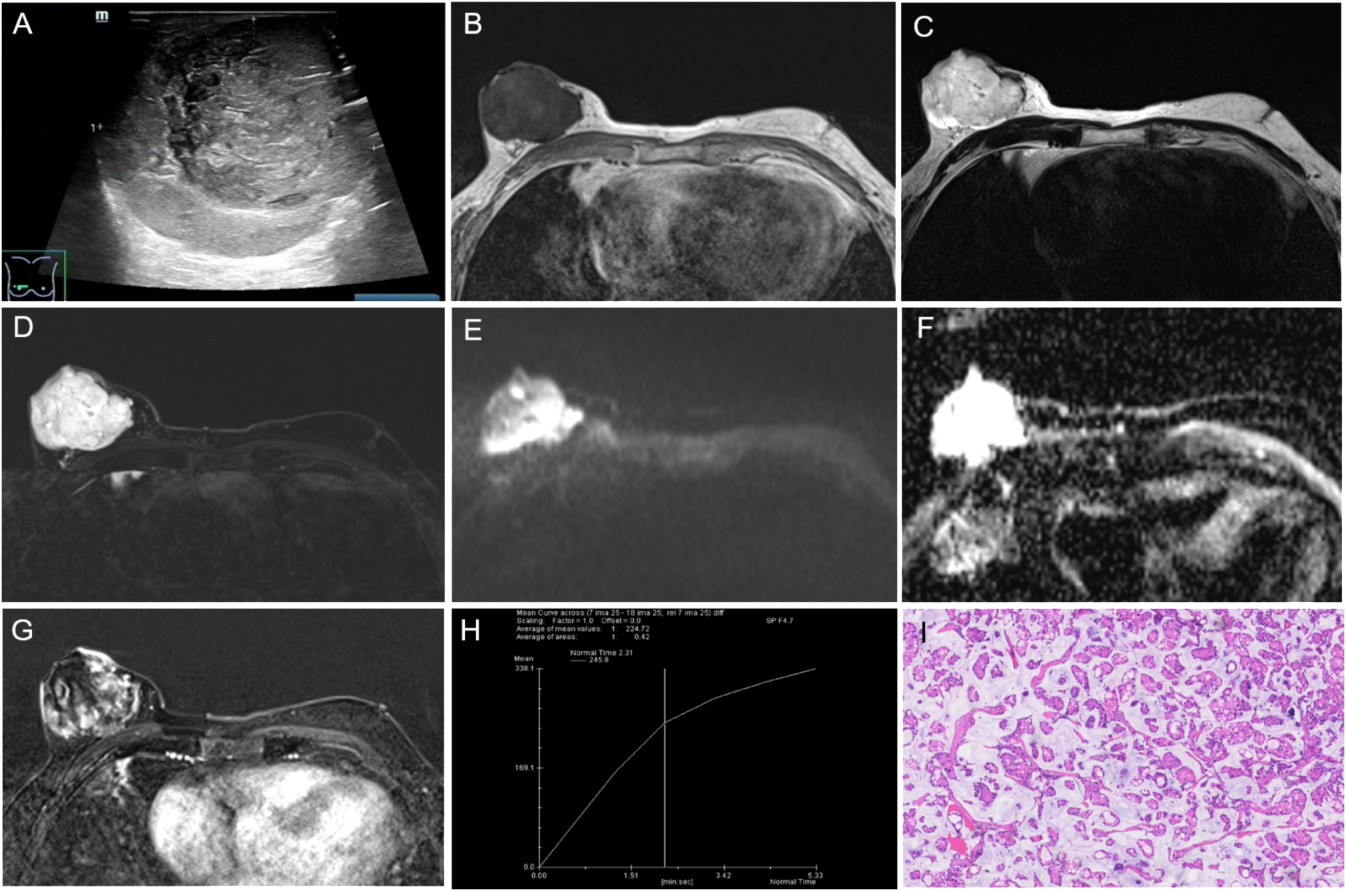
The results of imaging and pathological examinations. **(A)** Ultrasound showing a heterogeneous isoechoic mass behind the right breast nipple, with a lobulated shape and smooth edges. Breast MR images: **(B)** T1-weighted image (T1WI) demonstrating the mass with low signal intensity, **(C)** T2-weighted image (T2WI) and **(D)** fat-suppressed T2WI showing significantly high signal intensity. **(E)** Diffusion-weighted imaging (DWI) with a b-value of 800s showed high signal intensity. **(F)** the apparent diffusion coefficient (ADC) both showed high signal intensity. **(G)** The dynamic contrast-enhanced scan revealed the mass with heterogeneous, slow, and progressive enhancement. **(H)** The time-signal intensity curve (TIC) indicated type III (slow-persistent) enhancement. **(I)** Photomicrograph of histological image showing the tumor cells in trabecular, ribbon-like, and micropapillary shapes, containing a large amount of extracellular mucus (HE × 100). T1WI, T1-weighted image; T2WI, T2 weighted image; MRI, magnetic resonance imaging; DWI, diffusion-weightedimaging; TIC, time-signal intensity curve; ADC, apparent diffusion coefficient; HE, hematoxylin and eosin.

Ultrasound examination was performed on July 28, 2022. A heterogeneous isoechoic mass was observed behind the right nipple, approximately 60 mm × 43 mm in size, with a lobulated shape, smooth edges, a distinct capsule, and slightly increased posterior echoes. No lateral acoustic shadows were found (**Figure 2A**). Color Doppler flow imaging (CDFI) displayed no significant blood flow signals in the mass, while a small amount of blood flow signals was visible around the periphery. The mass was classified as BI-RADS 4B.

To further confirm the diagnosis, breast magnetic resonance imaging (MRI) was performed on August 8, 2022. The MRI showed the right breast exhibited increased volume, with a lobulated mass of approximately 48 mm × 56 mm × 56 mm in size and clear boundaries. The mass showed low signal intensity on T1-weighted image (T1WI) (**Figure 2B**), and high signal intensity on T2-weighted image (T2WI) (**Figure 2C**) and fat-suppressed T2WI (**Figure 2D**). Diffusion-weighted imaging (DWI) with a b-value of 800 s (**Figure 2E**) and apparent diffusion coefficients (ADCs) (**Figure 2F**) demonstrated high signal intensity. The dynamic contrast-enhanced scan revealed heterogeneous enhancement, with slow and progressive enhancement gradually filling the center of the lesion (**Figure 2G**). The time-signal intensity curve (TIC) was type III (slow-persistent) (**Figure 2H**). The right nipple displayed abnormal morphology, with indistinct boundaries near the tumor and evidence of adjacent skin invasion. The lesion was categorized as BI-RADS 6.

### Pathological and immunohistochemical results

Ultrasound-guided core needle biopsy (CNB) of the right breast mass was performed on August 4, 2022. The biopsy indicated three tissue strips with a mucinous appearance on the cut surface, revealing atypical cells in a mucus-rich background. The findings suggested the possibility of mucinous carcinoma.

The patient underwent breast surgery on August 11, 2022. Postoperative pathology: Gross pathology showed that the tumor’s cut surface was gray-white, gray-yellow, gel-like, with moderate texture and unclear boundaries. Microscopically, the tumor cells were trabecular, ribbon-like, and micropapillary, containing a large amount of extracellular mucus (**Figure 2I**). The cells had low-grade nuclei with rare nuclear division. Immunohistochemical results were summarized as follows: ER (approximately 70%, 2+), PR (approximately 60%, 2+), HER2 (1+), KI67 (approximately 5%+), E-Ca (+), P120 (+), P63 (-), Calponin (-), CK5-6 (-), EMA B (+), Syn (-), WY-1 (+). Based on the postoperative pathological results, the final diagnosis was mucinous carcinoma, classic type, grade II. The tumor invaded the skin of the areola papilla. Sentinel lymph node biopsy of the right axilla showed no signs of metastasis (0/3).

### Multidisciplinary expert consultation

A multidisciplinary consultation involving the departments of breast surgery, oncology, radiology, ultrasound, and pathology confirmed the diagnosis of primary mucinous carcinoma of the male breast based on histopathological examination and immunohistochemistry analysis of the excised tissue.

### Final diagnosis

The patient was diagnosed with pure mucinous adenocarcinoma of the male breast.

### Therapeutic intervention and follow-up

**Figure 3:**
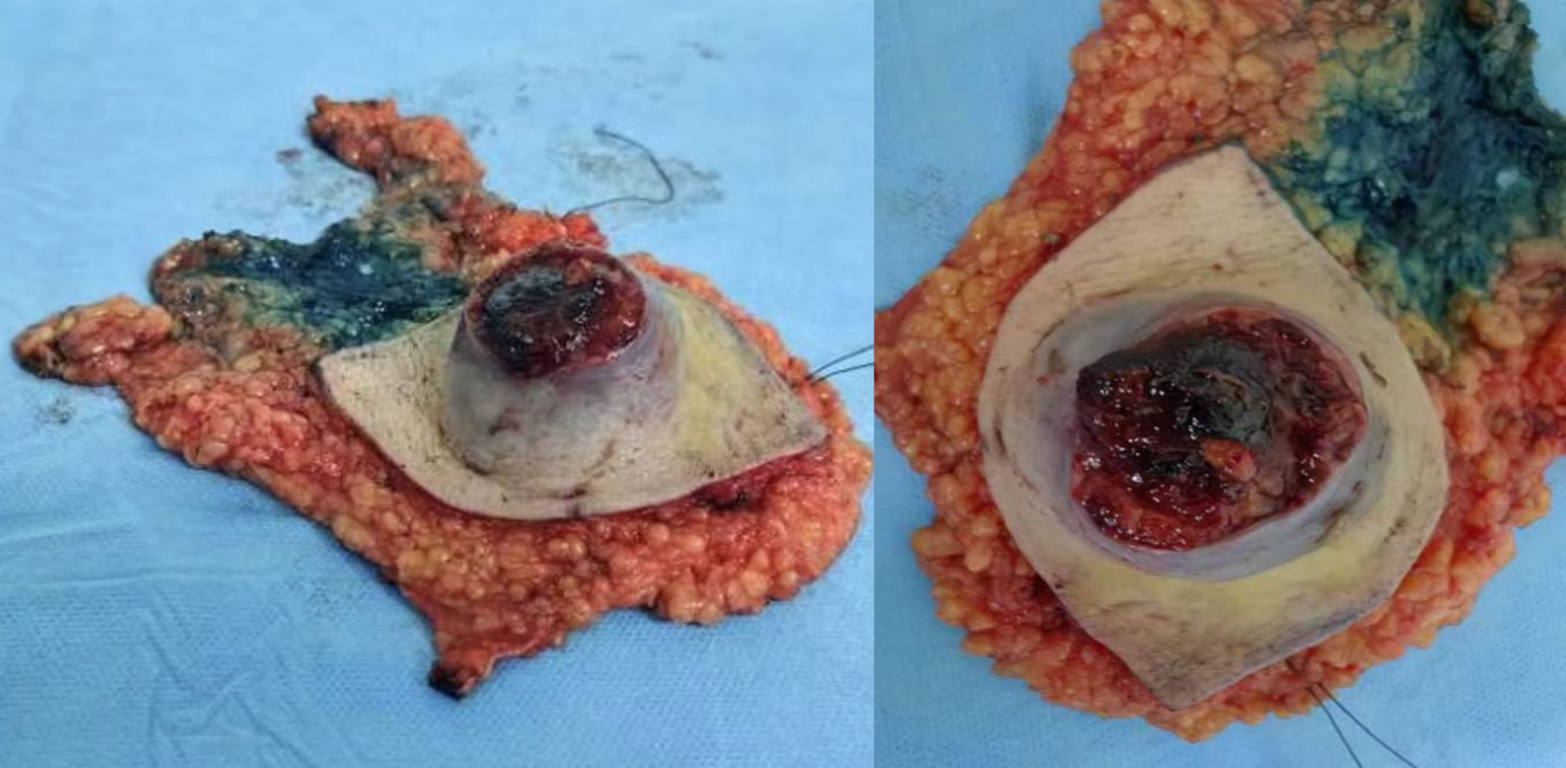
Right breast specimen.

The patient underwent modified radical mastectomy (**Figure 3**), followed by tamoxifen hormone therapy. He was monitored for two years, and no evidence of tumor recurrence was found.

## Discussion

Mucinous carcinoma, also referred to as colloid or gelatinous carcinoma, is among the rarest types of breast cancer, accounting for less than 2% of female breast carcinomas (3). Its occurrence in the male breast is extremely rare. Histopathologically, mucinous carcinoma is characterized by the presence of clusters of neoplastic cells suspended in extensive extracellular mucin, and it can be subclassified into pure and mixed types. Pure mucinous carcinoma type contains mucinous carcinoma components, accounting for more than 90% of the tumor, while the mixed type has both mucinous and invasive ductal carcinoma components (4). In the present case, diagnosis of pure mucinous carcinoma was made as no conventional invasive ductal carcinoma component was present. Pure mucinous carcinoma grows slowly, accompanying by relatively favorable prognosis, low recurrence rate, and low incidence of lymph node metastasis (5, 6). Its pathogenesis remains elusive, however, studies suggested its link with genetics and hormones (7, 8). Prior research demonstrated that gynecomastia is not a risk factor for male breast cancer (9).

Pure mucinous carcinoma is typically associated with favorable prognosis, and it is mainly misdiagnosed as a benign tumor on imaging examination (10). Even in female breasts, metastasis and invasion of contiguous structures are rare, typically presenting as a solitary breast mass without skin involvement or noticeable changes. However, in the present case, the tumor was found both in the skin and at the base of the areola and nipple. This could be attributed to the lower amount of fat in male breast tissue, which positions the tumor closer to the skin and increases the likelihood of dermal and basal infiltration. The histological and immunohistochemical characteristics of primary mucinous carcinoma of the skin and mucinous carcinoma of the breast overlap, complicating differential diagnosis. According to previous studies, three-quarters of mucinous carcinoma cases affecting the skin were found on the head, primarily on the eyelid (44%), with only a small proportion (5%) occurring on the chest (11, 12). Comparing the morphology and immune markers of the tumor in this case, it was difficult to determine whether it originated from the breast or chest wall skin. The primary site of the tumor indicates tissue origin: if the tumor begins on the skin surface, ruptures early, and then gradually grows into the breast, it is considered to originate from the skin appendages of the chest wall. Conversely, if the tumor originates deep within the breast tissue and subsequently involves the skin, it suggests a more typical breast origin. In the present case, the tumor was finally diagnosed as primary mucinous carcinoma of the male breast based on its primary site.

Pure mucinous carcinoma has imaging characteristics that may mimic benign lesions. On mammography, it mainly presents as a round or microlobulated, well-circumscribed high-density mass without microcalcification (10). On ultrasound, it appears as an isoechogenic mass with well-defined margins, relative to the surrounding fat, accompanying by posterior echo enhancement (13). MRI findings exhibit distinctive characteristics: on T1WI, signal intensity varies from low to high based on protein concentrations in the tumors. Mucinous carcinoma, owing to its abundant mucin content, typically demonstrates high signal intensity on T2WI (14). Early enhancement reveals gradual circular enhancement surrounding the tumor periphery, indicating that tumor cells are clustered around a central pool of mucin. The TICs from dynamic contrast-enhanced MRI exhibit a gradually enhancing pattern (15). Mucinous carcinoma needs to be distinguished from myxoid fibroadenoma, mucocele-like lesions, and encapsulated papillary carcinoma. Myxoid fibroadenoma and mucocele-like lesions present as distinct high-signal masses on T2WI, while they are mainly accompanied by coarse or amorphous calcification on mammograms. Encapsulated papillary carcinoma appears as a mixed solid and cystic mass with no enhancement of the cystic component and obvious enhancement of the solid component on enhanced scans. This case also needed differentiation of metastasis from mucinous adenocarcinoma of other sites, while no primary malignancy was found in this patient.

Although breast mucinous carcinoma and cutaneous mucinous carcinoma both belong to the category of mucinous carcinomas, there are some differences in their treatment methods. Primary mucinous carcinoma of the skin, a rare malignancy of the sweat glands, is characterized by low metastatic potential but a high recurrence rate. Lymph node dissection is typically unnecessary, as regional lymph node metastasis is rare in this type of carcinoma (16). Treatment concentrates on wide excision of the lesion, which may include standard local excision or Mohs micrographic surgery (17). Radiotherapy and chemotherapy are generally not utilized due to the resistance of cutaneous mucinous carcinoma to these modalities (18). In contrast, the standard treatment for male breast mucinous carcinoma involves modified radical mastectomy with sentinel lymph node biopsy, followed by adjuvant therapy. Hormonal therapy plays a pivotal role in treatment, given the high prevalence of hormone receptor positivity in male breast carcinomas (19). Patients with either type of carcinoma are recommended to undergo regular follow-up to monitor for local tumor recurrence or regional lymphadenopathy. However, standardized guidelines for the duration and frequency of follow-up sessions have not yet been provided (16, 20). The specific follow-up strategy should be based on the patient’s individual circumstances and physician’s advice.

Male breast mucinous carcinoma is rare and exhibits imaging characteristics similar to those of mucinous adenocarcinoma in female breast. However, distinguishing between breast mucinous carcinoma and primary mucinous carcinoma of skin on imaging is difficult due to overlapping histopathological features. Therefore, a definitive diagnosis mainly requires consideration of clinical history. Given the differences in clinical behavior and treatment strategies between mucinous carcinoma of the breast and primary mucinous carcinoma of the skin, it is crucial to ensure an accurate diagnosis.

## Data Availability

The original contributions presented in the study are included in the article/supplementary material. Further inquiries can be directed to the corresponding author.
